# Economic impact of homeopathic practice in general medicine in France

**DOI:** 10.1186/s13561-015-0055-5

**Published:** 2015-07-08

**Authors:** Aurélie Colas, Karine Danno, Cynthia Tabar, Jenifer Ehreth, Gérard Duru

**Affiliations:** 1 Laboratoires Boiron, Laboratoires Boiron, 2 avenue de l’Ouest Lyonnais, 69510 Messimy, France; 2 La-Ser Analytica and Conservatoire nationale des arts et métiers, Paris, France; 3 Cyklad Group, Lyon, France

**Keywords:** Conventional medicine, General practitioner, Homeopathy, Economic analysis, Prescribing practice

## Abstract

Health authorities are constantly searching for new ways to stabilise health expenditures. To explore this issue, we compared the costs generated by different types of medical practice in French general medicine: i.e. conventional (CM-GP), homeopathic (Ho-GP), or mixed (Mx-GP).

Data from a previous cross-sectional study, EPI3 La-Ser, were used. Three types of cost were analysed: (i) consultation cost (ii) prescription cost and (iii) total cost (consultation + prescription). Each was evaluated as: (i) the cost to Social Security (ii) the remaining cost (to the patient and/or supplementary health insurance); and (iii) health expenditure (combination of the two costs).

With regard to Social Security, treatment by Ho-GPs was less costly (42.00 € vs 65.25 € for CM-GPs, 35 % less). Medical prescriptions were two-times more expensive for CM-GPs patients (48.68 € vs 25.62 €). For the supplementary health insurance and/or patient out-of-pocket costs, treatment by CM-GPs was less expensive due to the lower consultation costs (6.19 € vs 11.20 € for Ho-GPs) whereas the prescription cost was comparable between the Ho-GPs and the CM-GPs patients (15.87 € vs 15.24 € respectively) . The health expenditure cost was 20 % less for patients consulting Ho-GPs compared to CM-GPs (68.93 € vs 86.63 €, respectively). The lower cost of medical prescriptions for Ho-GPs patients compared to CM-GPs patients (41.67 € vs 63.72 €) was offset by the higher consultation costs (27.08 € vs 22.68 € respectively). Ho-GPs prescribed fewer psychotropic drugs, antibiotics and non-steroidal anti-inflammatory drugs.

In conclusions management of patients by homeopathic GPs may be less expensive from a global perspective and may represent an important interest to public health.

## Background

The most effective ways to stabilise healthcare expenditure in France are widely debated by the authorities. Current healthcare represents approximately 10 % of all governmental spending (14.5 million €) [1]. The way in which this care is dispensed in general practice should be examined. The types of care practised and treatments prescribed could be evaluated with regard to their efficacy, usefulness, value to public health and economic impacts on society. This could help identify the most respectful and ethical practices, support the best use of medicines, obtain positive clinical outcomes and reduce costs. With this information, the health authorities could then make optimal choices.

Over half (56 %) of the French population has used homeopathic medicines and 11 % use these medicines regularly [2]. Homeopathic medicines are prescribed by general practitioners (GPs), but can also be recommended by and purchased directly from the pharmacy. In France, most GPs who prescribe homeopathic medicines undergo additional training on homeopathic medicine during their medical training or during their ongoing practice. The EPI3-La-Ser study [3] found that 24 % of patients in France consulted GPs who regularly or occasionally prescribed homeopathic medicines.

The evaluation of medical practices that prescribe homeopathic medicines is particularly important for public-health policy makers as it may have a considerable impact on healthcare costs. Although there have been many studies on the cost-efficacy of homeopathic or non-conventional treatments compared to other medical treatments [4–8], relatively few studies have analysed the actual costs of primary care to the Social Security Agency (l’Assurance Maladie), supplementary health insurance and the patient. In France, knowledge of the management of morbidity in hospitals has increased with the establishment of the PMSI (Programme de médicalisation des sytèmes d’information). This is not the same process that occurs in the ambulatory sector apart from some observational studies of general medicine and private company data from GP’s prescriptions taken from different surveys. However, the reimbursement of supplementary health insurance remains relatively poorly documented because there is a lack of publicly available data [9].

For example with respect to the potential of cost reduction, a study carried out in 2005 showed that complete replacement of the brand-name drug, Omeprazole (a proton pump inhibitor), by its generic counterpart for gastroesophageal reflux disease could reduce costs by 18.35 M€ (−4.3 % reimbursed expenditure) [10]. Another study showed that educational programs led to a reduction in antibiotic prescriptions given to children with acute nasopharyngitis, thus significantly reducing (−20 %) Social Security costs while following guidelines for care [11]. However, complementary healthcare’s contribution was not included in these studies.

As occurs in some other European countries and the US, homeopathic medicines are subject to management by the Social Security and the supplementary health insurances. To best understand French health insurance coverage, we compared the French data with those from the United States. French Social Security coverage is similar to that of the US Medicare system in terms of what is covered and what proportion of costs are covered. Any differences primarily lie in the type of populations covered. In France, individuals have their healthcare costs covered by a compulsory Social Security system. In this system, in 2008, the costs of medications were covered at 100 %, 65 %, 35 % or 0 % depending on drug-reimbursement rate in 2008. In addition to public insurance, people can also subscribe to supplementary insurance to obtain more complete coverage for their treatments. In France, there is a variety of private supplementary healthcare options, such as paying a standard fee-for-services or having a health care contingency account similar to those in the US. Today, 96 % of the French population are covered by supplementary health insurance, either individually, collectively through their employer, or through other free targeted public health insurance programmes (CMU-C) [12].

With 8,559 patients and 825 participating physicians, the EPI3 La-Ser study has been the largest study carried out to date in France to describe and compare, in a large representative sample of patients consulting their GPs, factors associated with different types of medical practice [13–18]. We used data from this study to examine the economic perspectives. This current-economic analysis was carried out on the population from the cross-sectional sample of the EPI3 study. In France, the gate keeper coordinates all of the patients’ care. He/she orients it if necessary towards a specialist clinician or a hospital service. The GP provides the first access to healthcare and has an overall view of the health status of the patient. As patients presenting with chronic musculoskeletal disorders and who consult with a homeopathic physicians state that their GP is their regular GP in 54.1 % of cases [15]. Thus to reduce bias, it was important in this study to only include patients who were visiting their regular GP in our analyses. This approach better reflects the patient’s healthcare management rather than that of any physician’s prescribing behaviour.

The aim of this study was to compare the costs generated by the different prescribing practices of the GPs (conventional, mixed or homeopathic) regarding all diseases that present in general practice. The different costs were compared from the point of view of Social Security, supplementary health insurance and the patients’ out-of-pocket expenditures. A description and a comparison of the drugs prescribed in the different medical practices was conducted.

## Methods

### Study design and participants

The EPI3 study was a French pharmaco-epidemiological study with a follow-up of 1-year that included a representative sample of GPs and their patients between March 2007 and July 2008 [3, 13–18].

The GPs, chosen at random from the national registry of French GPs, were invited to participate in the study. Recruitment was stratified according to the prescription preferences of the GPs, who were classified into three groups: (i) physicians who prescribed conventional medicines only (CM-GPs); these GPs declared that they had never or rarely used homeopathic or complementary or alternative medicines; (ii) GPs who prescribed homeopathic,complementary or alternative medicines regularly in a mixed practice (Mx-GPs); and (iii) registered homeopathic GPs (Ho-GPs) who prescribed mainly homeopathic medicines. The participating physicians answered a telephone questionnaire in order to allocate them to one of these three groups. During a second stage of sampling, a one-day survey of all patients attending the medical practice of each participating GPs was conducted by a trained research assistant. They included all patients seen on the single day of the study unless the patient’s health state precluded them from completing a self-administered questionnaire. The study was approved by the Commission Nationale Informatique et Libertés (CNIL) and the Conseil National de l’Ordre des Médecins (CNOM). The participating GPs received remuneration for their time whereas the patients did not.

The population analysed in our study included patients of all ages from the EPI3 study who had consulted with their regular GP at inclusion. Thus, the population studied is different to that in the cross-sectional EPI3 study because only patients who consulted their regular GP were selected. The large number of participating GPs and patients included favoured a fair representation of clinical practice in primary French healthcare. A previous analysis of the EPI3 survey showed that the distribution of GP’s individual characteristics differed only slightly from those published in French national statistics [19].

In terms of the patients’ representativeness, the sample of the EPI3 survey was compared with other nationwide studies and demonstrated its efficiency through criteria reported in the previous study. For instance, patients registered by health insurance as eligible for the disease of long duration (DLD; Affection Longue Durée ALD in French) programme accounted for 28 % of patients in the EPI3 survey, which is a similar percentage to the 27 % reported in the national census of GPs’ patients [20].

### Data collection

The data collected in the cross-sectional EPI3 study are described elsewhere [13–15]. Briefly, the descriptive variables of the patients were: age, gender, professional group (when the patient was <18-years the professional status of one of his/her parents was recorded), supplementary health insurance coverage, governmental universal health insurance coverage, type of GP consulted, length of follow-up by the GP, the main reason for consultation, hospital admissions during the previous 12 months, presence of a DLD (because additional governmental coverage was available for these individuals), a physical score on the SF12 scale and a mental score on the SF12 scale.

The variables concerning the physicians’ prescriptions were as follows: the medicines, phytotherapy (plant derived therapy) and/or oligotherapy (trace mineral derived) products, absences from work (duration of sick-leave, if there was a prescription), other medical examinations (radiological, CT scans, MRI, biological, non-hospital nursing care, and physiotherapy) and if patients were referred to a specialist.

### Criteria used for the economic evaluation

We evaluated the economic impact of medical consultations and medical prescriptions, according to the calculation used to estimate healthcare costs for long-term and non-hospital care, as described by Ehreth [21]. Hence, costs such as hospital admissions, which are relatively rare and were not the focus of this study, were excluded.

Three types of costs were analysed: (i) the cost of the consultation, using the tariffs for medical consultations for the 2008 period; (ii) the cost of medical prescriptions, involving the total amount of medicines prescribed using the prices listed in the Gers Officine January 2008 database [22]; and (iii) the total cost of management which combined the costs of consultations plus prescriptions.

According to the system of healthcare management in France, each of these costs was evaluated as the: (i) cost to Social Security; (ii) the remaining cost (to the patient and/or to the supplementary health insurance); and (iii) the health expenditure (a combination of the two previous costs).

Certain characteristics of the patients were taken into account in the calculation of the cost of medical prescriptions and consultations including DLD status, possession or not of supplementary health cover and age. For patients with a DLD, Social Security bears 100 % of the contractual cost regarding care and medicines related to the pathology. The cost of consultation took into account a lump sum payment of 1 € per consultation for patients aged >18-years. The cost of the consultation also took into account the sector designation of the GP. In France, 99 % of private GPs are covered by governmental tariffs, and they adhere to a medical contract, which is a signed agreement between Social Security and one or more medical associations. Sectors 1 and 2 are different types of medical contract. GPs in sector 1 implement the baseline tariffs laid down by Social Security and those in sector 2 charge whatever rates they like [23]. The average rate of exceeded fees for GPs in sector 2 was 44 % [24]. Social Security bears 70 % of the contractual cost.

A GP can choose to not adhere to the medical contract with Social Security (1 % of private GPs) and freely implement their tariffs, their fees are not reimbursed for their patient, and only their prescriptions are supported. The cost of a consultation with these GPs, placed in a sector termed “Not under agreement” could not be counted. Otherwise, the cost of consultation was set at 22 € for GPs in sector 1 and at 32 € for GPs in sector 2 (2008 prices).

The costs of medicines were attributed according to the name of the drug, its dosage and its duration. Analyses were conducted using the unit price of the drug, the number of prescribed boxes, and the Social Security reimbursement rate of the drug in 2008 (100 %, 65 %, 35 % or 0 %), with these data having been previously validated by experts.

The costs of the prescriptions were calculated by taking into account a medical exemption of 0.25 € because, according to the Commission des Comptes (in June 2009 [25]), one box of medicines out of two involves a medical exemption of 0.50 €.

The cost to the patient and to the supplementary health insurance was the cost remaining after reimbursement by Social Security. Disassociation of the two costs was not possible because the conditions of the supplementary health insurance contracts differed from one patient to another.

### Statistical analysis

Quantitative variables were described using the following parameters: group size, mean, standard deviation, median, 1st and 3rd quartiles, minimum, maximum and number of missing data. Qualitative variables were described according to their frequency, percentage and also the number of missing data.

Percentages were calculated for each sample without missing data. The evaluation criteria were compared using the chi-squared test for the qualitative variables and analysis of variance for quantitative variables. The costs were compared using analysis of variance. An overall *p*-value for the model (comparing all three groups) was obtained. Each group was then compared to the reference group (CM) and the respective *p*-values are shown. The level of significance of the statistical tests was set at 5 %.

All statistical analyses were carried out using SAS version 9.3 software.

## Results

### Characteristics of the study population

**Fig. 1:**
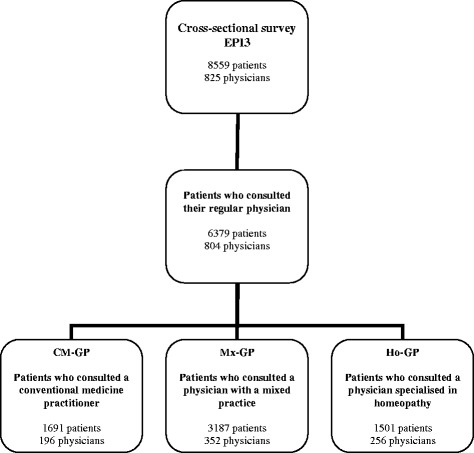
Flow-chart of the population analysed

The population analysed in this economic study consisted of 6,379 patients from the cross-sectional EPI3 study who consulted their gate keeper GP at inclusion (Fig. 1). This population was divided into three groups depending on the type of practice carried out by the GP: 1,691 patients saw a CM-GP, 187 patients consulted a Mx-GP and 1,501 patients saw a Ho-GP.

The socio-demographic characteristics of the patients in relation to the type of GP consulted are described in the original article on the EPI3 study [3]. Briefly, patients who consulted Ho-GPs were comparable in terms of age to those who consulted CM-GPs. However, Ho patients were more often female and had supplementary health insurance (*p* < 0.05). Ho patients less often had a DLD: 18.7 % vs 29.5 % for CM patients. Ho patients also had a better physical component when quality of life (QoL) was assessed with the SF12 scale (47.2 [SD: 10.6] vs 45.2 [SD: 11.1], respectively). However, these patients had a slightly lower score for the mental component of the SF12 scale than patients in the CM group (40.9 [SD: 10.5] vs 41.6 [SD: 10.9], respectively). No differences were observed in QoL scores between the CM and Mx patients. The Characteristics of treating physicians are also described in the published EPI3 study [3] . It was noteworthy that there were significantly more GPs in sector 2 in the Ho group than in the CM group (49.3 % vs 6.9 %, respectively; p < 0.05) and, therefore, there were significantly more GPs in sector 1 in the CM group than in the Ho group. Mx-GPs accounted for 94.6 % and 4.9 % in sectors 1 and 2, respectively. Only six Ho-GPs and one Mx-GP chose the sector termed “Not under agreement”: this included 32 patients from the Ho group (2 % of Ho-GP patients) and 17 patients from the Mx group (0.5 % of Mx-GP patients).

**Fig. 2:**
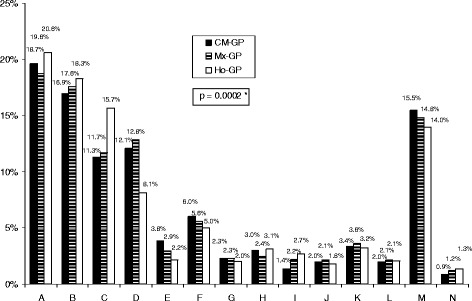
Distribution of the main reasons for consultation according to the type of medical practice (CM-GP, Mx-GP, Ho-GP). A: Diseases of the respiratory system including otolaryngology; B: Diseases of the musculoskeletal system; C: Neurotic disorders and sleep disorders; D: Cardiovascular and metabolism diseases; E: Endocrine diseases; F: Diseases of the digestive system; G: Diseases of the genitourinary system; H: Diseases of the nervous system, head and neck; I: Skin and subcutaneous tissues disorders; J: Infectious diseases and systemic parasitic diseases; K: Injury and poisoning; L: Pregnancy, postpartum, newborn, child; M: Patterns of use of health services (code V); N: Other diseases. GP, general practitioner; CM, conventional medicine; Mx mixed conventional and homeopathic practice; Ho homeopathy

Figure 2 shows the main reasons for a consultation as reported by the GPs. Overall, there was little difference between the three groups. A higher prevalence of anxiety and depressive problems, and sleep disorders and upper respiratory tract infections were observed in patients who consulted a Ho-GP compared to the CM group. For the CM patients, there was a higher prevalence of cardiovascular problems.

### Treatments prescribed at inclusion

Non-medicinal prescriptions (biological examinations, CT scans, sick-leave) were not evaluated in terms of cost but are described in Table 1. Patients in the Ho group had significantly fewer sick-leave episodes (8 % vs 11 % in the CM and Mx groups, respectively; *p* = 0.0051). However, the duration of these sick-leaves was comparable: 12 days for patients in the Ho group vs 11 days for CM patients and 13 days for Mx patients (*p* = 0.4128).

**Table 1:** Non-medicinal prescriptions

	CM group	Mx group	Ho group	*p*
	(*N* = 1691)	(*N* = 3187)	(*N* = 1501)	
Prescription of sick-leave, n (%)				
Yes	175 (11.2)	336 (10.8)	112 (7.9)	
No	1389 (88.8)	2783 (89.2)	1297 (92.1)	0.0051
Prescription of a radiological examination, n (%)				
Yes	104 (6.6)	183 (5.9)	79 (5.6)	
No	1463 (93.4)	2937 (94.1)	1328 (94.4)	0.4503
Prescription of a CT scan, n (%)				
Yes	16 (1.0)	28 (0.9)	7 (0.5)	
No	1538 (99.0)	3093 (99.1)	1392 (99.5)	0.2552
Prescription of MRI, n (%)				
Yes	16 (1.0)	28 (0.9)	8 (0.6)	
No	1535 (99.0)	3087 (99.1)	1390 (99.4)	0.3765
Prescription of a laboratory test, n (%)				
Yes	247 (15.7)	390 (12.5)	195 (13.9)	
No	1322 (84.3)	2736 (87.5)	1209 (86.1)	0.0084
Nursing care, n (%)				
Yes	14 (0.9)	29 (0.9)	7 (0.5)	
No	1539 (99.1)	3086 (99.1)	1393 (99.5)	0.3090
Prescription of physiotherapy, n (%)				
Yes	97 (6.2)	180 (5.8)	106 (7.6)	
No	1461 (93.8)	2939 (94.2)	1294 (92.4)	0.0698
Patient referred to a specialist, n (%)				
Yes	191 (12.4)	311 (10.0)	133 (9.5)	
No	1354 (87.6)	2792 (90.0)	1261 (90.5)	0.0201

*CM* conventional medicine; Mx mixed conventional and homeopathic practice; Ho homeopathy

Table 2 shows the quantity of prescriptions dispensed by the GPs. On average, patients in the Ho group had more medicines prescribed than patients in the CM or Mx groups (*p* < 0.0001), with two-times more non-reimbursable medicines prescribed and three-times more medicines reimbursable at 35 % (*p* < 0.0001). Ho-GPs prescribed two times less reimbursable medicines at 65 % (*p* < 0.0001).

**Table 2:** Description of prescriptions

Type of prescription	CM group	Mx group	Ho group	*p*
	(*N* = 1691)	(*N* = 3187)	(*N* = 1501)	
Prescriptions containing only homeopathic medicines, n (%)	1 (0.1)	49 (1.8)	342 (24.9)	<0.0001
Prescriptions containing only non-homeopathic medicines, n (%)	1345 (91.1)	2310 (83.4)	397 (28.9)	<0.0001
Mixed prescriptions, n (%)	8 (0.5)	157 (5.7)	440 (32.0)	<0.0001
Mean [SD] no. of drugs per prescription	3.1 [2.1]	2.8 [1.9]	3.5 [2.2]	<0.0001
Mean [SD] no. of non-reimbursable drugs per prescription	0.2 [0.4]	0.2 [0.5]	0.3 [0.6]	<0.0001
Mean [SD] no. of drugs with 35 % reimbursement rate per prescription	0.7 [0.9]	0.8 [1.1]	2.0 [1.9]	<0.0001
Mean [SD] no. of drugs with 65 % reimbursement rate per prescription	2.2 [1.9]	1.9 [1.6]	1.1 [1.5]	<0.0001

For the first three lines, prescriptions containing only phytotherapy and/or oligotherapy and/or radiological examinations do not appear. *SD* standard deviation

*CM* conventional medicine; *Mx* mixed conventional and homeopathic practice; *Ho* homeopathy

Three main categories of specific conventional medicines were prescribed by the GPs: i.e. for diseases of the respiratory system including otolaryngology; for diseases of the musculoskeletal system; and for neurotic disorders and sleep disorders (Table 3). These three main reasons for a consultation represented 49.5 % of all consultations (*n* = 6295). Homeopathic practitioners prescribed fewer non-steroidal anti-inflammatory drugs (NSAIDs) (−50 %) and antibiotics (−46 %) for respiratory tract infections, fewer analgesics (−66 %) and NSAIDs (−49 %) for musculoskeletal disorders, and fewer psychotropic drugs (anxiolytics, hypnotics and antidepressants) (−46 %) for anxiety-depressive disorders and sleep problems than CM-GPs.

**Table 3:** Specific conventional medicines prescribed for the three main categories of reasons for consultation

Type de medicines	CM group	Mx group	Ho group	*p*
	(*N* = 1691)	(*N* = 3187)	(*N* = 1501)	
A: Diseases of the respiratory system including otolaryngology				
At least one analgesic or NSAID (N02 and M01)	590 (34.9)	1013 (31.8)	261 (17.4)	<0.0001
At least one nasal preparation (R01)	158 (9.3)	277 (8.7)	50 (3.3)	<0.0001
At least one antibiotic (J01)	186 (11.0)	341 (10.7)	90 (6.0)	<0.0001
B: Diseases of the musculoskeletal system				
At least one analgesic (N02A and N02B)	123 (7.3)	200 (6.3)	37 (2.5)	<0.0001
At least one NSAID (without ASA) (M01A)	286 (16.9)	479 (15.0)	129 (8.6)	<0.0001
C: Neurotic disorders and sleep disorders				
At least one psychotropic drug (N03, N05, N06)	512 (10.4)	778 (9.1)	294 (5.7)	<0.0001
At least one anxiolytic (N05B)	162 (9.6)	245 (7.7)	77 (5.1)	<0.0001
At least one hypnotic or sedative (N05C)	81 (4.8)	121 (3.8)	42 (2.8)	0.014
At least one antidepressant (N06A)	166 (9.8)	248 (7.8)	74 (4.9)	<0.0001

All values shown are n (%)

*CM* conventional medicine; *Mx* mixed conventional and homeopathic practice; *Ho* homeopathy

*NSAID* non-steroidal anti-inflammatory drug

### Total cost of treatment strategies

Table **4** shows the total cost of treatment including the cost of the consultation and the cost of medical prescriptions.

**Table 4:** Total cost of treatment strategy (cost of the consultation + cost of the prescription)

	CM group (*N* = 1691)	Mx group (*N* = 3187)	Ho group (*N* = 1501)	*p* ^①^
Social Security cost in €, mean [SD]	65.25	60.51	42.00**	<0.0001
	(97.38)	(165.85)	(70.30)	
Cost of the consultation	16.49	16.21*	15.81**	<0.0001
	(2.97)	(2.82)	(2.55)	
Cost of the prescription	48.68	43.87	25.62**	<0.0001
	(96.32)	(162.74)	(68.23)	
Cost to patient and/or supplementary health insurance in €, mean [SD]	21.35	21.08	26.89**	<0.0001
	(29.24)	(28.12)	(27.79)	
Cost of the consultation	6.19	6.27	11.20**	<0.0001
	(3.88)	(3.60)	(5.75)	
Cost of the prescription	15.24	15.28	15.87	0.7917
	(28.15)	(27.74)	(27.25)	
Health expenditure (¥) in €, mean [SD]	86.63	81.60	68.93*	0.0034
	(106.97)	(170.04)	(81.53)	
Cost of the consultation	22.68	22.49	27.08**	<0.0001
	(2.53)	(2.17)	(5.0)	
Cost of the prescription	63.72	59.03	41.67**	<0.0001
	(106.28)	(167.29)	(79.74)	

CM, conventional medicine; Mx mixed conventional and homeopathic practice; Ho homeopathy

(¥) Health expenditure = Social Security cost + cost to patient and/or supplementary health insurance

***p*** ^①^= Overall *p*-value for the model (comparing all three groups). SD: standard deviation

* = significant *p*-values for comparisons between Mx vs CM and Ho vs CM; * = *p* <0.05; ** = *p* <0.0001

With regard to Social Security, treatment by Ho-GPs was less expensive (42.00 € vs 65.25 € for CM-GPs; *p* < 0.0001). Medical prescriptions were two-times less expensive to Social Security for patients in the Ho group (25.62 € vs 48.68 € for CM patients; *p* < 0.0001). The cost of the consultation was less expensive for patients in the Ho group (15.81 € vs 16.49 €; *p* < 0.0001) but was comparable in terms of value.

For the complementary health insurance and/or the patients’ out-of-pocket expenses, treatment by CM-GPs was less expensive due to the lower cost of consultations (6.19 € vs 11.20 € for the Ho group; *p* < 0.0001). However, the remaining cost of the prescriptions was comparable between the two groups of patients (15.87 € vs 15.24 € in CM patients; *p* = 0.7717).

The global cost was 20 % less expensive for patients in the Ho group than for those in the CM group (68.93 € vs 86.63 €, respectively; *p* = 0.0021). When the total cost was broken down into its two constituent parts, the total cost of the consultation was significantly lower for patients in the CM group than for those in the Ho group (22.68 € vs 27.08 €, respectively; *p* < 0.0001), whereas the cost of the medical prescription was significantly lower for patients in the Ho group (41.67 € vs 63.72 € for patients in the CM group; *p* < 0.0001).

## Discussion

The methodology used in the original cross-sectional study, which was designed to limit the bias often associated with pharmaco-epidemiological studies, has been presented previously [13]. Our comparison of the three types of management may help authorities in the current climate to reduce/scale down the reimbursement of medicines. Our results show that, for Social Security, the costs related to management by Ho-GPs were lower than those for GPs with a conventional practice.

However, the costs of consultations were higher for patients in the Ho group than in the CM group (27.08 € vs 22.68 €, respectively) possibly because there were more sector 2 physicians in the Ho group and, therefore, a higher level of unregulated overspending on consultancy fees (49.3 % of Ho patients vs 6.9 % of CM patients vs 4.9 % of Mx patients consulted a GP in sector 2). Thus, this imbalance in distribution of GP contracts had an impact on global cost. It should be noted that, in France, like in many other countries, the choice of regular physician is left to the patient. The consultation with a Ho-GP often lasts longer than a consultation with a CM-GP. This may reflect the fact that Ho-GP often see more patients who have a chronic disease [15], which could explain the imbalance in distribution of GP contracts.

The type of management is also interesting in terms of public health because Ho-GPs prescribed fewer medicines associated with possible misuse. Antibiotics, NSAIDs and psychotropic drugs are associated with important side-effects, including dependency or addiction for some classes of psychotropic drugs. Misuse or overuse of antibiotics can lead to the development of resistant bacteria and thus reduce their efficacy [26, 27]. In our study, as Ho patients used fewer NSAIDs, antibiotics and psychotropic drugs and therefore avoided the need for other treatments used to decrease these side-effects, they also avoided the associated supplementary costs.

Three cohort studies have been carried out using data from the original cross-sectional study. About 50 % of the patients included in the cross-sectional study were followed-up for 1 year measuring the impact of different methods of management on clinical outcomes, QoL, the side-effects and the medical care needed according to three main groups of diseases encountered in general medicine: i.e. musculoskeletal pain, sleep problems or anxiety/depression, upper respiratory tract infections. With regard to all the results published to date for two of these cohorts (i.e. upper respiratory tract infections, musculoskeletal pain), at 1 year, there was no evidence for any difference in terms of clinical benefit to the patients treated by the different types of GP [16, 18]. Regarding the cohort with sleep problems or anxiety/depression, the results also leant towards the same conclusions (article submitted). These cohort results show that integrating homeopathic medicines into prescriptions made by GPs does not seem to represent a loss of opportunity for the patients in terms of expected clinical outcomes. These results agree with the findings of Kooreman and Baars [28] who reported that patients who consulted GPs with additional complementary and alternative medicine training, including homeopathy, had lower healthcare costs than those who consulted conventional GPs.

There is a real public health interest in what patients resort to as first-line treatment from a Ho-GP. The cost to the patient is reasonable for an equivalent benefit to that for patients consulting CM-GPs.

Other studies in France have shown that Ho-GPs are less likely to prescribe antibiotics. For children aged 18 months to 4 years and who present with recurrent acute rhino-pharyngitis, Ho-GPs prescribed fewer antibiotics, the patients had fewer infectious episodes and complications, fewer sick-leaves taken by their parents and a better QoL, for direct medical costs to Social Security that were comparable to CM-GPs [29, 30]. In Europe, other studies have confirmed this trend in cost. A study carried out in 2003 showed that homeopathic medicines were less expensive than conventional medicines in the UK [31]. Another study showed that patients receiving homeopathic treatment had better results overall in terms of severity of symptoms, compared to patients receiving conventional treatment, whereas the total cost of the two groups was comparable [32].

The main strengths of the EPI3 study have already been acknowledged elsewhere. These include its representativeness drawn from a large national sample of physicians and patients, which minimized the risk of a selection bias. Moreover, no selection criteria were used according to health conditions or motives for consultation in order to closely reflect a typical consultation day in primary care.

However, the study has several limitations inherent to pharmaco-epidemiologic studies.

The physicians’ self-declarations of their frequency of use of complementary medicines or alternative medicines and homeopathy was used to classify the three groups. and this strategy has been shown to reflect real-life clinical practice. Although these definitions potentially limit the generalisation of our results as they represent the practice in France, we believe they can be compared to other practices in Europe and the US. Non-economic data and data on medical prescriptions (particularly for NSAIDs, antibiotics and psychotropic drugs) can be compared with data from other countries.

The costs of non-drug prescriptions such as for sick-leave were only measured in physical units and showed that sick-leave was significantly lower in patients treated by Ho-GPs compared to CM-GPs (8 % vs 11 %, respectively; *p* = 0.0051). These costs, not measured in terms of economic value, may be significant to Social Security, supplementary health insurance and the patient’s out-of-pocket expenses, and may also have an impact on the employer or business in general. The cross-sectional EPI3 study was a “photograph” of a given day of medical practice and the prescriptions written by GPs in France. Daily prescriptions were a mix of new prescriptions and renewals, and represented the patient’s status at one point in time. Due to its design, the study did not provide longitudinal clinical data that could measure relations such as cost/efficacy. However, the three cohort studies carried out on about 50 % of the patients included in our study provide data on clinical status.

We did not assess home consultations, which could have led to underestimation of the burden of disease and, therefore, a greater cost to the CM group. Our study is limited because the severity of the pathologies, which was associated with the number of prescribed medicines and therefore with the costs, could not be assessed. We were also unable to analyse the variability of the physicians’ prescriptions for any one specific diagnosis: to obtain this, we would have had to refine the diagnosis code.

Finally, analyses were descriptive but the observed results reflected real life.

The description of the patients in our three groups of GPs highlighted some differences: compared to patients in the GP-CM group, patients that consulted a Ho-GP were more likely to be educated and female and were also more likely to have a healthier lifestyle (a lower body mass index (BMI) and less likely to smoke or consume alcohol). These patients’self-perceived health expressed as QoL had a slightly lower mental health status but a slightly better physical health status: these differences, thus, could represent a selection bias.

The impact of gender, age and QoL (SF12) on medical costs need to be assessed in a further study.

## Conclusion

Our study provides information on the economic impact of homeopathy regarding all diseases treated in general practice in France. Our results show that the management of patients by a homeopathic GP was less expensive from a global perspective, in terms of total management (taking into account the cost of a consultation and the cost of prescriptions): i.e. 68.93 € for patients seen by a Ho-GP vs 86.63 € for patients seen by a CM-GP; thus, representing a saving of 20 %.
